# Reply to Najjar, R.S. Comment on “Dyńka et al. The Ketogenic Diet and Cardiovascular Diseases. *Nutrients* 2023, *15*, 3368”

**DOI:** 10.3390/nu15204312

**Published:** 2023-10-10

**Authors:** Damian Dyńka, Katarzyna Kowalcze, Anna Charuta, Agnieszka Paziewska

**Affiliations:** Institute of Health Sciences, Faculty of Medical and Health Sciences, Siedlce University of Natural Sciences and Humanities, 08-110 Siedlce, Poland; damian.dynka24@gmail.com (D.D.); katarzyna.kowalcze@uph.edu.pl (K.K.); anna.charuta@uph.edu.pl (A.C.)

We thank Dr. Najjar for his interest [1] in our recent publication [2]. The aim of the publication is not to establish a hierarchy of nutritional models, nor to issue a verdict on the best or worst diet in the prevention and treatment of cardiovascular diseases (CVDs). Our work is based solely on a thorough analysis of the potential of the ketogenic diet in this respect, also considering the results of the latest meta-analyses, systematic reviews and randomized controlled trials (RCTs). All objections to the publications cited by us should be addressed to their authors.

The American Heart Association (AHA) does not reject the ketogenic diet. The ketogenic diet only does not meet the AHA Criteria of recommended dietary patterns. At the same time, a ketogenic diet can be healthy if it contains, among other components, non-starchy vegetables, nuts, fish and excludes products with added sugar [3]. So, the safety of the ketogenic diet depends on its composition. 

Below are the answers to each of the points raised:

(I) In our publication, we only referred to the fulfillment of the Bradford Hill causality criteria, referring to the publication by Ravnskov et al. [4]. We have no influence on any paradigm because that is not our role. We made it clear in the publication that LDL cholesterol is still officially recognized as one of the causes of CVD. We cited, among other documents, The Consensus Statement from the European Atherosclerosis Society Consensus Panel [5], information from the National Center for Chronic Disease Prevention and Health Promotion (NCCDPHP) [6], and the results of a meta-analysis in this area [7]. However, the aim of the publication was to assess the potential of the ketogenic diet in the context of many parameters related to CVD, not to assess the effect of LDL on CVD. 

(II) The role and importance of LDL are analyzed in (I). The cited studies on hypocaloric diets are not conclusive. In our publication, we referred to the highest-ranking evidence. The fact that the effect obtained is also affected by weight loss has been mentioned many times in the text. The argument about the high amount of saturated fatty acids in the ketogenic diet is common, but aside from the very question of the impact of saturated fatty acids, a ketogenic diet doesn’t have to be high in saturated fatty acids.

(III) In the article, we did not conclude that the ketogenic diet was generally more effective in reducing body weight than other diets. It is obvious that the caloric deficit has the primary influence on this. It is known that it is easier for some people to maintain and follow a ketogenic diet than, for example, a low-fat diet, e.g., due to less intense feeling of hunger, even despite weight loss [8,9,10]. Often, a low-carbohydrate diet can be even easier (or just as easy) to maintain than a low-fat diet [11,12]. The role of appetite in observing a diet is crucial, while KD shows a significant advantage in this respect compared to diets with higher carbohydrate content [13]. The claim that body weight can be reduced to a greater extent with fat restriction, citing a short-term study [14] is a misstatement. It is known that the caloric deficit plays a primary role. Short-term studies on the impact of the ketogenic diet are not reliable due to the occurrence of a period of adaptation of the body to the use of ketone bodies. This is related to the occurrence of negative symptoms, especially in the first days of using the ketogenic diet. However, this is not reflected in the long-term perspective [15]. Therefore, the effect of a ketogenic diet on weight loss is the same as that of a low-fat diet if a caloric deficit is maintained. This is confirmed by the latest publication by Aronica et al., in which a ketogenic and low-fat diets had the same effect on body weight over a 12-month period [16]. 

(IV) In a ketogenic diet the body does not use glucose (therefore its concentration is reduced) but instead uses ketone bodies (produced from fatty acids) as the main source of energy [17]. Therefore, it is not surprising that in the state of ketosis, glucose challenge will result in a significant increase of glycemia [18]. As in pregnancy, when physiological insulin resistance develops in order to conserve glucose for the fetus [19], in this case too, it is by no means a pathological reaction. The body induces a mild state of insulin resistance by conserving glucose for some cells. However, this condition is quickly reversible when carbohydrates are reintroduced into the diet [20,21,22,23]. This was confirmed almost 100 years ago [24]. At the same time, it is worth noting that a caloric deficit will play a fundamental role in combating insulin resistance [25].

In the context of the impact on the vascular endothelium, we devoted the entire chapter “5” describing the beneficial effect of mainly ketone bodies on this aspect. The fact is that there are no studies describing strictly the impact of a well-balanced ketogenic diet on endothelial function, which we emphasize in the publication. At the same time, however, studies on diets containing up to 45% of energy from carbohydrates cannot verify the effectiveness of a ketogenic diet, because such an amount of carbohydrates does not induce the state of ketosis [26]. In addition, the issue of the composition of the diet returns, which we emphasize again—it can be crucial.

The conclusions of the publication are based on the cited studies, including meta-analyses, systematic reviews, and randomized controlled trials. Our team does not issue a verdict on the use of the ketogenic diet in cardiovascular diseases. Our goal is not to suggest the use of a ketogenic diet in CVDs, but to thoroughly review the literature in this field. We have clearly emphasized and reiterate that there is a need for further research in this area. At the same time, we strongly emphasize that the composition of the diet is pivotal.

## References

[1] Najjar R.S. (2023). Comment on Dyńka et al. The Ketogenic Diet and Cardiovascular Diseases. *Nutrients* 2023, *15*, 3368. Nutrients.

[2] Dyńka D., Kowalcze K., Charuta A., Paziewska A. (2023). The Ketogenic Diet and Cardiovascular Diseases. Nutrients.

[3] Gardner C.D., Vadiveloo M.K., Petersen K.S., Anderson C.A.M., Springfield S., Van Horn L., Khera A., Lamendola C., Mayo S.M., Joseph J.J. (2023). Popular Dietary Patterns: Alignment with American Heart Association 2021 Dietary Guidance: A Scientific Statement from the American Heart Association. Circulation.

[4] Ravnskov U., de Lorgeril M., Diamond D.M., Hama R., Hamazaki T., Hammarskjöld B., Hynes N., Kendrick M., Langsjoen P.H., Mascitelli L. (2018). LDL-C does not cause cardiovascular disease: A comprehensive review of the current literature. Expert Rev. Clin. Pharmacol..

[5] Borén J., Chapman M.J., Krauss R.M., Packard C.J., Bentzon J.F., Binder C.J., Daemen M.J., Demer L.L., Hegele R.A., Nicholls S.J. (2020). Low-density lipoproteins cause atherosclerotic cardiovascular disease: Pathophysiological, genetic, and therapeutic insights: A consensus statement from the European Atherosclerosis Society Consensus Panel. Eur. Heart J..

[6] Hearth Disease and Stroke. https://www.cdc.gov/chronicdisease/resources/publications/factsheets/heart-disease-stroke.htm.

[7] Peng K., Li X., Wang Z., Li M., Yang Y. (2022). Association of low-density lipoprotein cholesterol levels with the risk of mortality and cardiovascular events: A meta-analysis of cohort studies with 1,232,694 participants. Medicine.

[8] Gibson A.A., Seimon R.V., Lee C.M., Ayre J., Franklin J., Markovic T.P., Caterson I.D., Sainsbury A. (2015). Do ketogenic diets really suppress appetite? A systematic review and meta-analysis. Obes. Rev..

[9] Valinejad A., Khodaei K. (2022). Does exercise during a ketogenic diet effectively alter appetite sensation, appetite-regulating hormones, and body composition?. Exp. Biol. Med..

[10] Napoleão A., Fernandes L., Miranda C., Marum A.P. (2021). Effects of Calorie Restriction on Health Span and Insulin Resistance: Classic Calorie Restriction Diet vs. Ketosis-Inducing Diet. Nutrients.

[11] Hession M., Rolland C., Kulkarni U., Wise A., Broom J. (2009). Systematic review of randomized controlled trials of low-carbohydrate vs. low-fat/low-calorie diets in the management of obesity and its comorbidities. Obes. Rev..

[12] Hull M., Leaf A., Brown W., Sicart P.A. (2019). Evidence Based Keto. Examine.com. https://tonygentilcore.com/wp-content/uploads/2019/12/Keto-Guide-Examine.com_.pdf.

[13] https://www.virtahealth.com/blog/ketosis-appetite-hunger.

[14] Hall K.D., Bemis T., Brychta R., Chen K.Y., Courville A., Crayner E.J., Goodwin S., Guo J., Howard L., Knuth N.D. (2015). Calorie for Calorie, Dietary Fat Restriction Results in More Body Fat Loss than Carbohydrate Restriction in People with Obesity. Cell Metab..

[15] Shalabi H., Alotaibi A., Alqahtani A., Alattas H., Alghamdi Z. (2021). Ketogenic Diets: Side Effects, Attitude, and Quality of Life. Cureus.

[16] Aronica L., Landry M.J., Rigdon J., Gardner C.D. (2023). Weight, insulin resistance, blood lipids, and diet quality changes associated with ketogenic and ultra low-fat dietary patterns: A secondary analysis of the DIETFITS randomized clinical trial. Front. Nutr..

[17] Puchalska P., Crawford P.A. (2017). Multi-dimensional Roles of Ketone Bodies in Fuel Metabolism, Signaling, and Therapeutics. Cell Metab..

[18] Rosenbaum M., Hall K.D., Guo J., Ravussin E., Mayer L.S., Reitman M.L., Smith S.R., Walsh B.T., Leibel R.L. (2019). Glucose and Lipid Homeostasis and Inflammation in Humans Following an Isocaloric Ketogenic Diet. Obesity.

[19] Leoni M., Padilla N., Fabbri A., Della-Morte D., Ricordi C., Infante M. (2022). Mechanisms of Insulin Resistance during Pregnancy. Evolving Concepts in Insulin Resistance.

[20] Caminhotto Rde O., Lima F.B. (2013). Impaired glucose tolerance in low-carbohydrate diet: Maybe only a physiological state. Am. J. Physiol. Endocrinol. Metab..

[21] Soeters M.R., Soeters P.B. (2012). The evolutionary benefit of insulin resistance. Clin. Nutr..

[22] Tzagournis M., Skillman T.G. (1970). Glucose intolerance mechanism after starvation. Metabolism.

[23] Kinzig K.P., Honors M.A., Hargrave S.L. (2010). Insulin sensitivity and glucose tolerance are altered by maintenance on a ketogenic diet. Endocrinology.

[24] Tolstoi E. (1929). The effect of an exclusive meat diet lasting one year on the carbohydrate tolerance of two normal men. J. Biol. Chem..

[25] Mason C., Foster-Schubert K.E., Imayama I., Kong A., Xiao L., Bain C., Campbell K.L., Wang C.Y., Duggan C.R., Ulrich C.M. (2011). Dietary weight loss and exercise effects on insulin resistance in postmenopausal women. Am. J. Prev. Med..

[26] Schwingshackl L., Hoffmann G. (2013). Low-carbohydrate diets impair flow-mediated dilatation: Evidence from a systematic review and meta-analysis. Br. J. Nutr..

